# Domestication is associated with increased interspecific hybrid compatibility in landfowl (order: Galliformes)

**DOI:** 10.1093/jhered/esad059

**Published:** 2023-09-28

**Authors:** James M Alfieri, Reina Hingoranee, Giridhar N Athrey, Heath Blackmon

**Affiliations:** Interdisciplinary Program in Ecology and Evolutionary Biology, Texas A&M University, College Station, TX, USA; Department of Poultry Science, Texas A&M University, College Station, TX, USA; Department of Biology, Texas A&M University, College Station, TX, USA; Department of Epidemiology and Biostatistics, Texas A&M University, College Station, TX, USA; Interdisciplinary Program in Ecology and Evolutionary Biology, Texas A&M University, College Station, TX, USA; Department of Poultry Science, Texas A&M University, College Station, TX, USA; Interdisciplinary Program in Ecology and Evolutionary Biology, Texas A&M University, College Station, TX, USA; Department of Biology, Texas A&M University, College Station, TX, USA

**Keywords:** artificial selection, bird, Haldane’s Rule, reproductive barriers, speciation

## Abstract

Some species are able to hybridize despite being exceptionally diverged. The causes of this variation in accumulation of reproductive isolation remain poorly understood, and domestication as an impetus or hindrance to reproductive isolation remains to be characterized. In this study, we investigated the role of divergence time, domestication, and mismatches in morphology, habitat, and clutch size among hybridizing species on reproductive isolation in the bird order Galliformes. We compiled and analyzed hybridization occurrences from literature and recorded measures of postzygotic reproductive isolation. We used a text-mining approach leveraging a historical aviculture magazine to quantify the degree of domestication across species. We obtained divergence time, morphology, habitat, and clutch size data from open sources. We found 123 species pairs (involving 77 species) with known offspring fertility (sterile, only males fertile, or both sexes fertile). We found that divergence time and clutch size were significant predictors of reproductive isolation (McFadden’s Pseudo*-R*^2^ = 0.59), but not habitat or morphological mismatch. Perhaps most interesting, we found a significant relationship between domestication and reproductive compatibility after correcting for phylogeny, removing extreme values, and addressing potential biases (*F*_1,74_ = 5.43, *R*^2^ = 0.06, *P*-value = 0.02). We speculate that the genetic architecture and disruption in selective reproductive regimes associated with domestication may impact reproductive isolation, causing domesticated species to be more reproductively labile.

## Introduction

Reproductive isolation and divergence time have a strong relationship across many clades, although the rate at which reproductive isolation accumulates varies widely. For example, while the most diverged species pair that can still hybridize and result in viable offspring in mammals is *Camelus dromedarius* (dromedary camel) and *Lama guanicoe* (guanaco) (~20 MY), in fish, it is *Acipenser gueldenstaedtii* (Russian sturgeon) and *Polyodon spathula* (American paddlefish) (~150 MY). In birds, the most diverged hybrid is between *Numida meleagris* (Helmeted guineafowl) and *Gallus gallus* (chicken) (51 to 65 MY) (Alfieri et al. 2023). To our knowledge, in plants, the most diverged hybrid is between *Gymnocarpium* sp. and *Cystopteris* sp. (~60 MY) (Rothfels et al. 2015). The evolutionary drivers of such differences in barriers to hybridization are poorly understood. Several factors can contribute to the variable accumulation of reproductive isolation, such as different rates of regulatory evolution among species. For example, mammals have faster rates of regulatory evolution than birds, which can cause greater probabilities of developmental incompatibilities (Prager and Wilson 1975; Fitzpatrick 2004). In this study, we investigated the relationship between reproductive isolation and divergence in Galliformes, an order of birds with vast ecological, physiological, and morphological divergences and with numerous domesticated species.

Domestication is an “evolutionary process that arises from a specialized mutualism, in which one species controls the fitness of another to gain resources and/or services” (Purugganan 2022). While some studies suggest intraspecies reproductive isolation between domesticated and wild populations is required in domestication (Ladizinsky 1985; Dempewolf et al. 2012), others have found extensive gene flow, questioning the necessity for reproductive isolation (Frantz et al. 2015). As far as we know, no prior works have characterized the influence of domestication on interspecies reproductive isolation. As some domestication traits contradict traits under natural selection, it raises the possibility that domestication may impede reproductive isolation. Domesticated organisms are often selected for traits favoring hybridization, such as circumventing female choice, altering the costs and benefits of mate choice, and decreasing paternal investment (Willis 2013). Another reason why domesticated organisms may be better at hybridizing is due to higher recombination rates compared with their wild counterparts (Otto and Barton 1997; Ross-Ibarra 2004). Indeed, higher recombination rates allow for faster uncoupling between deleterious and beneficial variants, which is also a characteristic of introgressed regions of the genome (Schumer et al. 2018).

On the other hand, we can imagine why domesticated species may rapidly accumulate reproductive isolation; domesticated species experience multiple genetic bottlenecks due to intense positive selection for production traits (Price 1984). As reproductive incompatibility is a trait that has segregating variation both within (Demuth and Wade 2007a,b; Cutter 2012) and between (Morejohn 1968) species, these bottlenecks could lead to the fixation of alleles causing reproductive incompatibilities. As domestication may lead to the breakdown or the reinforcement of reproductive barriers (or have no effect), we need a robust analysis of this interaction.

Reproductive isolation generally increases with divergence time in stages that align with Haldane’s Rule (Haldane 1922). Before divergence, individuals of a species are not typically postzygotic reproductively isolated. As divergence increases, individuals from the heterogametic sex will become infertile or inviable, followed by the homogametic sex. The genetic mechanisms that explain Haldane’s Rule include the Dominance Hypothesis (Turelli and Orr 1995; Orr and Turelli 1996; Zeng 1996), Meiotic Drive (Frank 1991; Hurst and Pomiankowski 1991; McDermott and Noor 2010), Faster X (Charlesworth et al. 1987; Coyne and Orr 1989), sex chromosome interactions (Turelli and Orr 2000), and faster heterogametic sex (Tao and Hartl 2003). Eventually, species will diverge until both sexes of the hybrid are sterile or inviable, a significant component of the Biological Species Concept (Dobzhansky 1935; Mayr 1963). Numerous studies have used comparative studies of reproductive isolation to examine the time course of Haldane’s Rule expression (Matute and Cooper 2021), including the avian order Galliformes (Price and Bouvier 2002; Arrieta et al. 2013).

Habitat divergence among species is one of the most well-investigated drivers of speciation (Rice 1984; Funk et al. 2006). Indeed, two of the most well-studied examples of sympatric speciation are based on habitat divergence. The walking-stick insect *Timema cristinae* has two ecotypes, each specializing in a host plant, *Ceanothus spinosus* or *Adenostoma fasciculatum* (Sandoval 1994). These two ecotypes have different color morphs, each displaying better crypsis on its host plant. Divergent selection for microhabitat (host plant) has promoted reproductive isolation between the two ecotypes (Nosil et al. 2002). The other classic example of sympatric speciation is the case of the apple maggot fly, *Rhagoletis pomonella* (Bush 1969). In this system, the ancestral host plant was the Hawthorn (*Crataegus* sp.). However, introducing the domesticated apple (*Malus pumila*) caused some *R. pomonella* populations to colonize it instead. Divergent selection on phenology has promoted reproductive isolation between the two ecotypes (Filchak et al. 2000). These examples illustrate habitat divergence as a critical mechanism for generating species boundaries.

Unlike habitat divergence, the relationship between morphological or physiological divergence and reproductive isolation is unclear. For example, cryptic species are not morphologically differentiated yet are reproductively isolated (Worsham et al. 2017). However, these cryptic species often diverge physiologically (Chen and Hare 2008; Dennis and Hellberg 2010; Muangmai et al. 2015). On the other hand, some species of insects can have exceptionally different genital morphologies but are not reproductively isolated (Masly 2012). And still, in other cases, morphological divergence (for example, body size in Centrarchid fishes (Bolnick et al. 2006) does promote reproductive isolation. However, despite numerous examples of divergent selection acting on morphology and physiology, divergent selection may not cause reproductive isolation.

Galliformes are a diverse bird order that includes many common clades like pheasants, turkeys, quails, grouse, guineafowl, and guans. The group diverged from the Galloanserae ancestor at the K-Pg boundary around 65 MYA and has radiated to over 290 extant species globally distributed with diverse phenotypes (Berv et al. 2023). Some species, such as the chicken (*G. gallus*), helmeted guineafowl (*N. meleagris*), and turkey (*Meleagris gallopavo*), have been domesticated and, as such, have experienced strong selection for production traits (e.g. meat and eggs). Other species, such as the peafowl (*Pavo cristatus*) and golden pheasant (*Chrysolophus pictus*), are captive-bred for ornamental aviculture. Others, such as the common pheasant (*Phasianus colchicus*) and Bobwhite Quail (*Colinus virginianus*), are captive-bred and reared for hunting. Due to their close association with humans, abundant records document wild and captive hybrids. These reasons make Galliformes an excellent model to examine the drivers of the accumulation of reproductive isolation. In this study, we investigated the role of divergence time, domestication, and mismatches in morphology, habitat, and clutch size among hybridizing species on reproductive isolation in Galliformes. We found that divergence time is the best predictor of reproductive isolation, and physiological divergence has a subtle but clear relationship with reproductive isolation. Additionally, we show that domestication is associated with increased interspecific hybrid compatibility.

## Methods

To catalog recorded instances of hybridization in Galliformes, we compiled and analyzed hybridization occurrences from literature and recorded measures of postzygotic reproductive isolation. We extracted and deduplicated all hybridization records in the families Phasianidae, Odontophoridae, Cracidae, Megapodiidae, and Numididae from the Serge Dumont Bird Hybrids Database (Dumont 2003). We collapsed all subspecies to the species level and deleted all records of hybridizations between subspecies but still within the same species. Additionally, we performed forward and backward literature searches for references within the Handbook of Avian Hybrids of the World (McCarthy 2006) and Bird Hybrids: A Checklist with Bibliography (Gray 1958). We attempted to access the original reports within these databases to confirm the accuracy of the information regarding the referenced cross, as Gray has been noted to include dubious crosses (Wu and Davis 1993; Alfieri et al. 2023). When present, we recorded F1 fertility information (sterile, only males fertile, and both sexes fertile). If a cross did not have recorded F1 fertility information, we excluded it from the analysis. We also collected the median divergence time estimate from the TimeTree database for each species pairing in our dataset (Kumar et al. 2017, 2022).

To examine the time course of Haldane’s Rule in Galliformes, we performed a logistic regression of the fertility of crosses as a function of the divergence time of each cross. Galliformes, like all birds, have a ZZ/ZW sex chromosome system, where the female is the heterogametic sex. Crosses that exhibited Haldane’s Rule (only males were fertile) scored zero, while those where both sexes were fertile scored one. We used the fitted model to determine the divergence time required for a hybridization event to have a 50% probability of exhibiting Haldane’s Rule. We performed an additional logistic regression between complete reproductive isolation and divergence time to examine the time course of speciation. Crosses that resulted in fertile offspring were scored as “0,” while crosses that resulted in inviable or sterile offspring scored “1.”

To determine the relative roles of time, habitat, morphological, and physiological divergence in driving reproductive isolation, we performed a generalized linear multiple regression between these variables and the reproductive isolation score of the hybrid offspring. For these analyses, the unit of replication is each hybridizing species pair. The reproductive isolation score of the hybrid offspring was scored as one if all offspring were sterile, two if only male offspring were fertile, and three if offspring of both sexes were fertile. We used habitat mismatch as a proxy of habitat divergence. We obtained habitats for our species from the AVONET database (Tobias et al. 2022). We scored species pairs as “1” if they occupied the same habitat type or “0” if they occupied different habitats. We obtained morphological data from the AVONET database (Tobias et al. 2022). The morphological variables included 11 variables: beak length culmen, beak length nares, beak width, beak depth, tarsus length, wing length, Kipps distance, secondary 1, hand wing index, tail length, and mass. We calculated absolute value differences between these morphological traits for each species pair, performed principal component analysis (PCA), and subsequently reduced the dimensions to the first two PCA axes, which explained 68% of the variance. We used clutch size difference to measure physiological divergence because clutch size is essential to reproductive energy allocation (Drent and Daan 1980). We obtained clutch sizes for a previous compilation (Jetz et al. 2008) and the Birds of the World collection (Winkler et al. 2020). We fit the generalized linear multiple regression using reproductive isolation score as the response variable, habitat mismatch, clutch size difference, and the two PCA axes of morphological differences as the predictor variables. We used R package stats (R Core Team 2022) and performed stepwise model selection using the Akaike information criterion (AIC) (Akaike et al. 1973).

To assess the impact of domestication, we first needed to calculate a species-specific reproductive isolation index. This score should capture whether a species accumulates reproductive isolation more quickly or slowly than typical for Galliformes. To calculate this score, we fit a simple linear model where the outcome of hybridization was the response variable and was scored as one if all offspring were sterile, two if only male offspring were fertile, and three if offspring of both sexes were fertile. The predictor variable for this model was the divergence time between the hybridizing species. Then, we calculated the mean of all residuals involving that species and assigned this mean as the reproductive isolation index for each species.

To determine the impact of domestication on the accumulation of reproductive isolation, we needed to generate a measure of the degree of domestication. We used a text-mining approach leveraging the Avicultural Magazine, taking the incidence of species in the magazine as an index of degree of domestication. This magazine has been published since 1894 and focuses on breeding and keeping birds in captivity. We downloaded all available issues (*N* = 152) from the Biodiversity Heritage Library. Next, we collected synonyms (collected from a variety of sources e.g. IOC, Clements, and Birdlife) for scientific and common names for all the species in our hybridization dataset. We then searched the entirety of Avicultural Magazine for each occurrence of all collected species names. We then manually corrected text strings that overlapped (e.g. turkey vs. ocellated turkey). Finally, we rescaled these raw counts to a score from zero to one. To ensure that a potential underestimation of the chicken domestication index did not impact our study, we repeated our analysis after artificially setting the chicken domestication index to the maximum value of one. We found no substantive effect on the results. We then fit a generalized linear model with the domestication index as the predictor variable and the reproductive isolation index as the response variable. We found that the residuals of this model had a significant phylogenetic signal, so we used phylogenetic generalized least squares using R packages caper and phytools (Revell 2012; Orme et al. 2018).

We recognize that using domesticated species in comparative analyses may affect our results by observation and publication bias (e.g. domesticated species being crossed with more diverged species and a bias toward publishing successful crosses). This bias should be ameliorated to some extent because our reproductive isolation index is a mean of residuals. However, to evaluate this potential publication bias, we used two approaches. The first approach was to test whether any species in Galliformes accumulates reproductive isolation more quickly or slowly than typical for the clade. If publication bias affected our results, we expect species with more records or a higher domestication index to have significantly higher mean residuals. We compared the reproductive isolation index of each species to a permutation-generated null distribution of residuals. We calculated the mean of an equal number of randomly drawn residuals as the focal species and repeated this process 10,000 times. Because we have 76 species in our dataset, we used a Bonferonni correction and considered tests significant at an alpha of 0.0004. The second approach to determining the effect of this potential bias was to test for an association between the number of observations per species and the mean residual. If this bias affected our results, we expect the number of observations to be positively correlated with the mean residual.

## Results

**Fig. 1.**
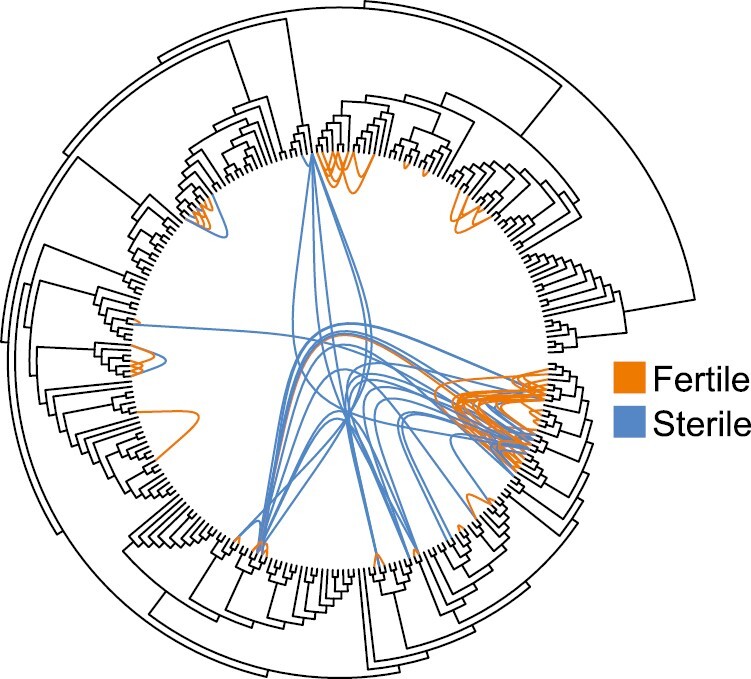
Hybridizations with documented offspring fertility in the bird order Galliformes. The orange links represent crosses where offspring (some or all) are fertile. The blue links represent crosses in which all offspring are sterile. The phylogeny is from the TimeTree database (Kumar et al. 2022)..

We found 123 species pairs (involving 77 species) with known offspring fertility (sterile, only males fertile, or both sexes fertile) (Fig. 1; Table S1). The five inter-family hybridizations all involved the helmeted guineafowl (*N. meleagris*) hybridizing with species from Phasianidae; these pairs had the maximum divergence time, 51 MY, and had sterile offspring. Among species pairs that retained only male fertility, the maximum divergence was 31 MY between *P. colchicus* and *G. gallus*. To determine whether or not this data point was driving our observed patterns, we performed all subsequent analyses with and without this data point. Removing this data point led to quantitative differences but no qualitative differences in results. For this reason all results we report below include this record. The mean divergence time among hybridizing pairs was 13.9 MY (CI: 1.5 to 26.3). The minimum divergence time was 0.105 MY between *Crossoptilon crossoptilon* and *C. harmani*. Some species may hybridize with many species, while others may only hybridize with few. The mean number of species that focal species have been recorded to hybridize with was 3.2 (CI: 0.1 to 6.3). *P. colchicus* had the greatest number of observations which had offspring fertility information (*n* = 15), followed by *G. gallus* (*n* = 13), *Lophura nycthemera* (*n* = 12), *L. leucomelanos* (*n* = 11), and *Syrmaticus reevesii* (*n* = 11).

**Fig. 2.**
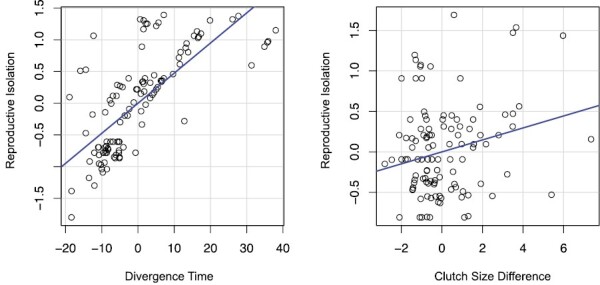
Added variable plots of the partial correlation between divergence and reproductive isolation and clutch size difference and reproductive isolation. Each circle represents one of the crosses, and the blue line is the line of best fit.

We analyzed the impact of various factors (habitat, clutch size, morphology) on reproductive isolation using regression and AIC model selection. The best model (AIC = 200.18, McFadden’s Pseudo-*R*^2^ = 0.59) used only divergence time (coefficient = 0.05, *P*-value = 0) and clutch size (coefficient = 0.07, *P*-value = 0.01) as predictor variables (Fig. 2).

**Fig. 3.**
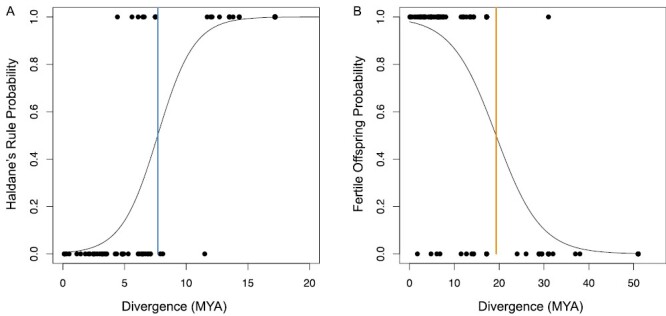
Logistic regressions of Haldane’s Rule (A) and the time to speciate (B). A) 1 represents hybrids where only the males are fertile, and 0 represents hybrids where both sexes are fertile. The blue line represents the divergence time where there is an equal probability of a cross resulting in a hybrid where both sexes are fertile or only the males are fertile. B) 1 represents crosses resulting in a hybrid with any fertility (both and only males), and 0 represents sterility of both sexes. The orange line represents the divergence time with an equal probability of a cross resulting in a sterile or fertile hybrid offspring.

To examine the time course of Haldane’s Rule, we performed a logistic regression using only observations where some offspring were fertile (*n* = 85). We found a 50% probability of observing Haldane’s Rule at 7.69 MY diverged (Fig. 3A). The most diverged cross that resulted in both sexes being fertile was between *Aplectoris barbara* and *Alectoris rufa*, which diverged 11.5 MYA. To examine the time course of speciation, we performed a logistic regression comparing observations of sterility and observations where at least one sex was fertile (*n* = 123). We found a 50% probability of species exhibiting complete reproductive isolation at 19.35 MY diverged (Fig. 3B). The least diverged species pair exhibiting complete reproductive isolation was *Callipepla californica* and *Callipepla gambelii* (1.77 MY diverged).

**Fig. 4.**
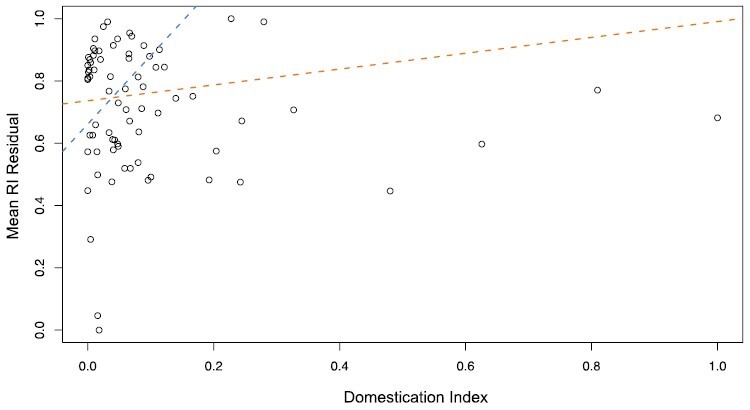
The phylogenetic generalized linear model between reproductive isolation and domestication index. The orange line represents the best fit of all data, while the blue line represents the best fit, excluding the 10 most and 12 least domesticated species.

To determine if domestication impacts the evolution of reproductive isolation, we fit a phylogenetic generalized least squares model between domestication and the mean residuals of a generalized linear model between reproductive isolation and divergence. We found a significant relationship between domestication and reproductive isolation (*F*_1,74_ = 5.438, *R*^2^ = 0.06, *P*-value = 0.02243) (Fig. 4). However, we note that this effect is relatively small in magnitude compared with the impact of divergence time. We repeated the analysis to determine if species with very high or low domestication values were driving this relationship, excluding the ten most and 12 least domesticated species. This difference (10 vs. 12) was because the 12 least domesticated species share the same score. These subsetted data show that the relationship between domestication and reproductive isolation is still significant and has a greater effect size (*F*_1,52_ = 14.33, *R*^2^ = 0.20, *P*-value = 0.0004).

**Fig. 5.**
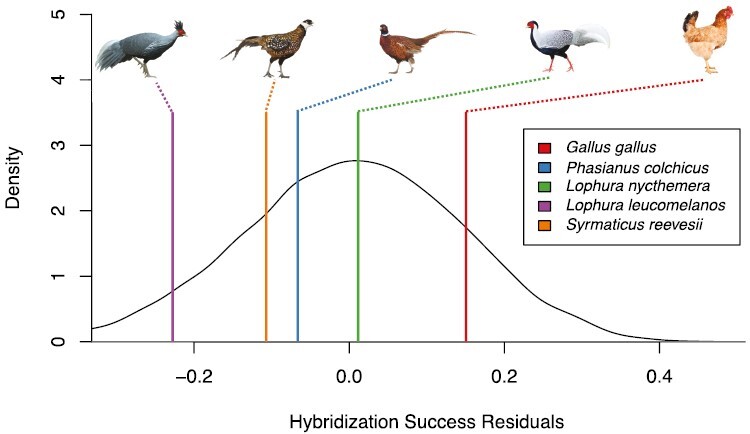
Mean residuals of the five species with the most hybridization records with documented offspring fertility plotted against a null distribution of 10,000 samplings of 13 species (*Gallus gallus*).

We found no publication bias impacting our inferences of the relationship between domestication and the reproductive isolation index. Our permutation-based approach to assess the differences in mean residuals between individual species showed that no species in our dataset had a reproductive isolation index significantly higher or lower than expected by chance (Fig. 5). Our second approach was to test the relationship between the number of observations of a species in our dataset and the mean residual for that species. We did not find a significant relationship (McFadden’s Pseudo-*R*^2^ = 0.006, *P*-value = 0.507).

## Discussion

In this study, we investigated the factors driving reproductive isolation in Galliformes and examined the role of domestication and other potential predictors in shaping these patterns. We found that divergence time and clutch size difference were the strongest predictors of reproductive isolation, outperforming other considered factors such as morphology and habitat mismatch. Our study supports previous findings that Galliformes follow Haldane’s Rule. Most interestingly, we found a significant relationship between domestication and reproductive isolation after correcting for phylogeny, removing extreme values, and addressing potential biases. This research contributes to understanding the complex dynamics underlying reproductive isolation in Galliformes and highlights critical factors influencing speciation processes.

### Predictors of reproductive isolation

We assessed the roles of habitat, morphology, clutch size, and divergence time in predicting reproductive isolation in Galliformes and found that only divergence time and clutch size were significant predictors. Clutch size is one of the most well-studied life-history traits, reflecting an evolutionary trade-off between extrinsic factors like resource availability and intrinsic factors like energetic constraints (Jetz et al. 2008). Interspecific variation in clutch size is dictated by coadapted gene complexes and physiological constraints like basal metabolic rate, field metabolic rate, and endocrine control mechanisms (Ricklefs and Wikelski 2002). Decreased clutch sizes have been observed in grosbeak F1 hybrids, and it has been suggested that this could be an example of postzygotic isolation acting on female hybrid fitness (Anderson and Daugherty 1974; Mettler and Spellman 2009). Additionally, in great tits, divergent great tit populations on the island of Vlieland in the North Sea have differing clutch sizes with a major genetic component. The clutch size divergence is maintained through differential immigration from mainland populations, not due to the divergent selection on clutch size itself (Postma and van Noordwijk 2005). However, to our knowledge, clutch size difference has not been directly tested for an association with reproductive isolation in birds and only has been characterized in relatively few other systems (Wiwegweaw et al. 2009; McBride and Singer 2010; Singer and McBride 2010; Belk et al. 2020). One of the best examples is from Orr (1996), where he measured fitness across a grasshopper hybrid tension zone, performed mate choice assays, and associated variation in life-history traits, including clutch size. He found that clutch size was associated with hybrid fitness across the cline, among other variables. He hypothesized that the genes determining clutch size might have a pleiotropic effect on reproductive isolation.

Habitat mismatch and morphological divergence were not significant predictors of reproductive isolation. Recently, Anderson and Weir (2022) tested whether trait differences between allopatric pairs of vertebrate species evolved due to adaptive divergence, drift, or similar selective pressures toward similar trait optima. They found that morphological differences between allopatric species pairs did not evolve due to adaptive divergence but due to adaptive evolution to similar selective pressures. As habitat mismatch was also not a significant predictor of reproductive isolation, divergent ecological selection may not be an essential factor driving reproductive isolation in Galliformes.

Many comparative studies testing the relationship between divergence and reproductive isolation have been conducted (reviewed in Matute and Cooper 2021), including in Galliformes (Price and Bouvier 2002; Arrieta et al. 2013). In a similar study to ours, Arrietta et al. (2013) correlated continuous data of offspring viability (percentage of unhatched eggs and proportion of male hybrids) and sterility (percentage of unfertilized eggs) to genetic divergence (percentage sequence divergence in COI and cytb) for F1 hybrids, F2 hybrids, and backcrosses. Although the nature of our data is different (they use continuous measures of hybrid inviability and sterility, while we only categorize hybrids as both sexes being fertile, male-only fertile, or sterile), our results do support their work. Together they provide a more complete assessment of Haldane’s Rule expression in Galliformes, in that Haldane’s Rule applies to both viability and fertility. Their study found that the proportion of male hybrids was greater in all hybrid generations, indicating that males have greater viability than females. Likewise, we found that hybrid males have greater fertility than females.

The time course of speciation greatly varies among clades, however, quantitatively characterizing these differences is challenging because not all studies have used time-calibrated phylogenies. Using time-calibrated phylogenies, we found that Galliformes evolve full intrinsic postzygotic isolation exceptionally slowly, at around 51 MY divergence. In Centrarchid fishes, full intrinsic postzygotic isolation is not realized until 28 MY (Bolnick and Near 2005; Coughlan and Matute 2020). In passerine birds, this time course is around 3 to 5 MY divergence (Price and Bouvier 2002; Coughlan and Matute 2020). We performed a post hoc comparison of our results with a recent study in mammals (Allen et al. 2020). Repeating our analysis of their mammal data (converting cytb divergences to TimeTree median divergences (Kumar et al. 2022), we found that the divergence time at which we would predict a 50% probability of observing Haldane’s Rule (females only fertile) was 5.40 MY (*n* = 13), compared with our estimate for Galliformes of 7.69 (*n* = 85). Previous studies have confirmed that hybrid inviability evolves much faster in mammals than in birds and suggested that this was due to faster regulatory evolution in mammals (Prager and Wilson 1975; Fitzpatrick 2004). As male sterility evolves faster than female inviability in Galliformes (Arrieta et al. 2013), we can speculate that different rates of regulatory evolution also cause greater divergence times of observing Haldane’s Rule in Galliformes than in mammals.

### Domestication

Domestication is an evolutionary process that involves the selection for traits that make species more amenable to human management, such as increased docility and enhanced productivity. Our analysis indicates a covariation of the degree of domestication and the rate that reproductive isolation accumulates, and this relationship is not affected by extreme domestication values. We speculate that domestication may impact reproductive isolation in two ways: disruption in selective reproductive regimes and domestication’s genetic architecture.

In a recent review, Gleeson and Wilson (2023) proposed that disruption in reproductive regimes results in common trait changes across species, commonly termed “domestication syndrome.” Their framework suggested that this could occur through altered sexual selection affecting mate choice (e.g. decreased choosiness) and altered reproductive niches affecting physiology (e.g. reduced seasonality and increased stress tolerance). These same selective pathways may also explain the relationship between domestication and reproductive compatibility. In natural settings, Galliformes exhibit a variety of mating strategies, including lekking, monogamy, and polygyny (Guan et al. 2022). However, under domestication, humans have typically controlled mate choice. As a result, the selective pressures that maintain these traits in wild populations are relaxed in domestic populations, leading to a decline in the expression of mate choice behaviors that maintain species boundaries.

Interestingly, a previous analysis of reproductive isolation in crops suggested that reproductive isolation may be viewed as a domestication trait (Dempewolf et al. 2012). However, this analysis only considered reproductive isolation between species and their wild counterpart (commonly but imprecisely termed progenitor) and found that domestication should increase this reproductive isolation. Our results suggest the opposite—that domestication should decrease reproductive isolation and that reproductive compatibility may be viewed as a domestication trait. We speculate that “reproductive tolerance” may result from the increased ability to cope with a broader set of environmental conditions and tolerate stress, which is critical for domestication in Galliformes (Jensen 2014).

The human-mediated diversification of distinct breeds is a hallmark of domestication (Larson and Fuller 2014). As breeds are selected for divergent phenotypes, they acquire phenotypic and genetic differences encompassing a wide range of traits, including morphology, behavior, and reproductive characteristics. This increased intraspecific variation can lead to a greater trait overlap between the breeds of the diversified species and individuals of another species, resulting in increased reproductive compatibility (Pfennig and Rice 2014; Shizuka and Hudson 2020). Although the existence and potential effects of domestication bottlenecks are debated (Muir et al. 2008; Frantz et al. 2015; Bosse 2019; Brown 2019), we know that a reduction in population size and strong multilocus selection that accompanies domestication may lead to indirect selection for increased recombination rates, a characteristic that is widely accepted to facilitate introgression (Burt and Bell 1987; Ross-Ibarra 2004; Li et al. 2019). Domesticated animals often show a correlated suite of traits (e.g. change in fur or plumage color, decreased brain size, altered endocrine responses, reduced fear, etc.), known as the domestication syndrome (Jensen 2006). As tameness is one of the requirements for domestication, it has been hypothesized that quantitative trait loci (QTL) for tameness overlap with QTL for other domestication syndrome traits (Agnvall et al. 2018). Additionally, a single gene under selection for a domestication trait may have pleiotropic effects (Wright 2015). Further, QTL for different traits may be part of the same regulatory network, and an upstream change may affect many downstream genes associated with reproductive isolation (Trut et al. 2004; Wright 2015). In Galliformes, QTL affecting domestication may have pleiotropic effects impacting reproductive incompatibility.

One weakness of our data is that we cannot determine whether domestication has impacted reproductive isolation or if these traits covary with some unmeasured trait in our dataset. It may be the case that species with greater propensities to hybridize are also the species that are easier to domesticate. Another limitation of our data is that we cannot tease apart postcopulatory mate choice from postzygotic isolation—postcopulatory sexual selection (e.g. cryptic female choice and sperm competition) is an important mechanism in some Galliformes (Tan et al. 2017; Pizzari and McDonald 2019). Another limitation is that we ignore intraspecific variation in reproductive isolation and instead assign a single trait value to a species. Indeed, Dobzhansky-Muller incompatibilities can be polymorphic within populations, and this information can be used to tease apart the relative contributions of drift and selection (Demuth and Wade 2007a; Cutter 2012; Kozlowska et al. 2012). However, despite these limitations, our study provides insight into how certain traits are associated with interspecies variation in reproductive isolation.

A species is a hypothesis that an evolutionary trajectory will remain perpetually diverged. We remain unaware of the extent of human impact on the evolutionary trajectories of the natural world. In this study, we found that animal domestication, an anthropogenic evolutionary process affecting many clades of life, may lead to the evolution of increased reproductive compatibility between diverged species. Further studies are needed to disentangle the direction of effect (if reproductively labile species make good domesticates or if domestication causes reproductive lability). Future studies should test this relationship in different clades with variable domestication and known hybridization outcomes (e.g. mammal orders Artiodactyla and Carnivora). Additionally, future studies should examine the impact of domestication pathways (commensal vs. prey vs. directed) on this relationship (Zeder 2012). Finally, future studies should incorporate measures of intraspecific variation in reproductive isolation, as this may be a vital contributor to interspecific variation in reproductive isolation. Despite these limitations, our findings provide insight into the evolutionary processes driving speciation in Galliformes.

## Supplementary Material

esad059_suppl_Supplementary_Table_S1

## Data Availability

All data and scripts necessary to repeat our analyses are available at https://github.com/JamieAlfieri/galliformeshybridsynthesis.
